# The utility of apoptosis inhibitor of macrophages as a possible diagnostic marker in patients with Crohn’s disease

**DOI:** 10.1186/s12876-017-0591-z

**Published:** 2017-03-11

**Authors:** Yohei Ono, Shuji Kanmura, Yuko Morinaga, Kohei Oda, Katsuto Kawabata, Shiho Arima, Fumisato Sasaki, Yuichirou Nasu, Shiroh Tanoue, Shinichi Hashimoto, Hiroki Taguchi, Hirofumi Uto, Hirohito Tsubouchi, Akio Ido

**Affiliations:** 1 Digestive and Lifestyle Diseases, Kagoshima University Graduate School of Medical and Dental Sciences, 8-35-1, Sakuragaoka, Kagoshima, 890-8520 Japan; 2 Center for Digestive and Liver Diseases, Miyazaki Medical Center Hospital, Miyazaki, Japan; 3 Kagoshima City Hospital, Kagoshima, Japan

**Keywords:** Inflammatory bowel diseases, Crohn’s disease, Macrophages, Apoptosis inhibitor of macrophages

## Abstract

**Background:**

Apoptosis inhibitor of macrophages (AIM) was initially identified as an apoptosis inhibitor that supports the survival of macrophages against various apoptosis-inducing stimuli, and AIM produced by macrophages may contribute to the pathogenesis of inflammatory bowel diseases (IBDs). However, there have been no reports on the kinetics of AIM in IBD and the impact of AIM on the pathogenesis of IBD. In this study, we aimed to investigate the diagnostic utility of levels of AIM and their correlation with the activity of Crohn’s disease (CD) and IBD.

**Methods:**

We used an enzyme-linked immunosorbent assay (ELISA) to examine AIM serum levels in 16 healthy subjects and 90 patients with inflammatory bowel diseases, namely 39 with CD and 51 with ulcerative colitis (UC), as well as 17 patients with Behcet’s disease (BD) as intestinal disease controls. We compared serum AIM levels among groups and examined whether there were correlations between serum AIM levels and disease activity and type. We also performed immunohistochemical staining of AIM in intestinal tissues of patients with CD.

**Results:**

Serum AIM levels were significantly higher in patients with CD than in patients with UC, BD, and controls (3.27 ± 2.14, 1.88 ± 1.43, 2.34 ± 1.37, and 2.13 ± 0.64 μg/ml, respectively; *P* < 0.01). There was no difference in serum AIM levels before and after treatment in patients with CD. However, in these patients the diagnostic rate using AIM was better than that based on anti-*Saccharomyces cerevisiae* antibodies. AIM was expressed in macrophages that were positive for CD14, CD16, or both in the intestinal tissues of patients with CD.

**Conclusions:**

AIM is a novel biomarker of CD that can distinguish CD from UC or BD. It is suggested that AIM may contribute to intestinal inflammation by inhibiting the apoptosis of macrophages.

## Background

Inflammatory bowel diseases (IBDs), including Crohn’s disease (CD) and ulcerative colitis (UC), are chronic intestinal disorders of unclear etiology [1]. The incidence of both conditions is increasing globally, and it is suggested that disease risk correlates with environmental changes [2]. Although the etiology of IBD remains unknown, it is thought to be strongly associated with the microbiome and the environmental factors that influence the microbiome [3]. Intestinal macrophages influence local homeostasis and help maintain a balance between commensal microbiota and the host. Additionally, intestinal macrophages play essential roles in intestinal inflammation. As a result, disorders of intestinal macrophages may cause unbalanced immune responses to commensal bacteria and lead to the development of chronic intestinal inflammation [4]. These macrophages play an important role in the pathogenesis of IBDs such as CD [5, 6].

The diagnosis of IBD is based on endoscopic, histologic and radiologic criteria. Disease activity in IBD is determined using both direct and non-invasive laboratory markers. However, endoscopy is still the gold standard diagnostic test, even though it is invasive [7, 8]. Several serological markers, such as C-reactive protein (CRP), as well as erythrocyte sedimentation rate (ESR) and platelet count or fecal calprotectin have been reported to be useful for diagnosing IBD and assessing disease activity and response to therapy [9–15]. An ideal marker for IBD would have high disease specificity, predictive ability for relapse of disease activity, and is cheap and noninvasive [16]. Currently, several serologic markers have been suggested as useful for the diagnosis, differentiation, and better comprehension of the pathogenesis of IBD [17–20]. Among them, anti-*Saccharomyces cerevisiae* antibodies (ASCA) and perinuclear antineutrophil cytoplasmic antibodies have been reported to be useful serum biomarkers for CD and UC, respectively [21]. ASCA has a low positivity rate of between 38.3 and 45.6% in CD patients, but there is low sensitivity and specificity for the diagnosis of CD [22, 23]. A definite diagnosis is necessary for properly choosing clinical follow-up and suitable therapies. However, some IBD cases share several endoscopic and histologic features with both CD and UC; these are defined as indeterminate colitis [24]. Therefore there is still a need in clinical settings for IBD markers with high sensitivity and specificity.

Apoptosis inhibitor of macrophages (AIM) was initially identified as an apoptosis inhibitor that supports the survival of macrophages against various apoptosis-inducing stimuli [25]. AIM is produced exclusively by tissue macrophages and itself influences these cells [26]. Higher blood levels of AIM are associated with autoimmune disease, but only in obese individuals. Arai et al. implicated blood AIM as a biomarker for obesity-associated autoimmune disease in humans [27]. Disorders of intestinal macrophages may lead to the development of chronic intestinal inflammation, and AIM produced by the macrophage may contribute to the pathogenesis of IBDs such as CD [4–6]. However, there have been no reports on the kinetics of AIM in IBD and the impact of AIM on the pathogenesis of IBD. In this study, we aimed to investigate AIM kinetics in the blood and tissues of patients with IBD, and examined the utility of AIM as a biomarker of IBD. We then determined whether blood levels of AIM were associated with clinical features such as disease activity and type.

## Methods

### Patients

This study enrolled 90 IBD patients, 39 of whom were diagnosed with CD and 51 with UC (Table 1). Seventeen patients with intestinal Behcet’s disease (BD) were used as intestinal disease controls. There were also 16 healthy individuals in the control group. Of the 39 patients with CD, 20 were men and 19 were women, with a mean age of onset of 32.4 years (range, 8–79 years). Of the 51 patients with UC, 27 were men and 24 were women, with a mean age of onset of 45.2 years (range, 8–79 years). Of the 17 patients with BD, 9 were men and 8 were women, with a mean age of onset of 47.5 years (range, 8–79 years). The mean age of healthy controls was 32.4 years (range, 8–79 years). All IBD patients were diagnosed using established endoscopic, radiological, histological, and clinical criteria. The clinical characteristics of subjects are summarized in Table 1. Twelve patients with CD had high disease activities, defined as a Crohn’s Disease Activity Index (CDAI) score of 150 or greater. Clinically, CD occurred as the ileal type in 9 patients, the ileocecal type in 25 patients, the colonic type in 4 patients, and unknown type in 1 patient. Twenty-two patients with UC were in the remission phase while 29 were in the active phase. The active phase was defined as a Lichtiger’s clinical activity index (CAI) score greater than or equal to 6. All patients with BD had high disease activity based on clinical symptoms. Blood samples were collected with informed consent. The patients were treated at Kagoshima University Hospital from January 2007 to December 2013. The sera and intestinal tissue specimens of patients were stored at −80° until analysis. Excised intestinal tract tissue was stored after formalin fixation. Serum analysis was performed using the reagents and kits mentioned below. The ethics committee at Kagoshima University Hospital approved the study protocol, and written informed consent was obtained from each participant.

**Table 1:** Clinical characteristics

	CD	UC	BD	Control	*P* value
Number of patients	39	51	17	16	
Gender					
Male	20	27	9	14	0.070
Female	19	24	8	2	
Mean age (±SD), yr	32.4 ± 14.5	45.2 ± 22.8	47.5 ± 17.6	32.4 ± 3.8	0.014
BMI (±SD), kg/m^2^	19.0 ± 3.6	21.6 ± 3.7	20.4 ± 3.8		0.006
CDAI					
≥ 150	12				
< 150	27				
CAI					
≥ 6		29			
< 6		22			
Disease location					
CD					
Ileal type	9				
Ileocolonic type	25				
Colonic type	4				
Unknown	1				
UC					
Pancolitis		41			
Left side colon		4			
Rectum		5			
Unknown		1			
Current treatment					
5-ASA	25	43	13		
Steroid	2	18	6		
Azathioprine	0	5	0		
Infliximab	5	0	0		
Adalimumab	1	0	0		
GMA/LCAP	1	6	1		
AIM (μg/ml)					
Average (±SD)	3.3 ± 2.1	1.9 ± 1.4	2.3 ± 1.4	2.1 ± 0.6	<0.001
WBC (±SD), x10^2^/μl	6.7 ± 2.6	8.4 ± 3.9	7.3 ± 2.8	–	0.055
CRP (±SD), mg/dl	2.5 ± 3.5	3.6 ± 5.8	2.7 ± 4.0	–	0.929
IgM (±SD), mg/dl	141.5 ± 124.3	118.0 ± 77.9	118.0 ± 64.9	–	0.275

*Abbreviations*: *GMA/LCAP* granulocyte and monocyte adsorptive apheresis / leukocytapheresis, *WBC* White blood cell, *CRP* C-reactive protein, *IgM* Immunoglobulin M

### ELISA measurements of serum AIM and ASCA levels

We measured the serum AIM concentrations in 90 patients with IBD, 17 with BD, and 16 heathy individuals as described above. AIM levels were determined using ELISA kits from Trans Genic Inc. (Kumamoto, Japan). For comparison, we measured ASCA immunoglobulin G (IgG) in patients with CD and UC, but not those with BD. ASCA IgG concentrations were measured using ELISA kits from Genesis Diagnostics (Cambridgeshire, UK). Briefly, serum samples diluted at 1:100 were added to wells and incubated for one hour at room temperature, and then each well was washed three times with phosphate-buffered saline. Horseradish peroxidase-conjugated anti-human IgG was added to each well.

### Immunohistological analysis

Small intestinal tissues from patients with active-phase CD were obtained after ileocecal resection due to intractability to medical treatment. Briefly, tissue samples were fixed with 10% buffered formalin, embedded in paraffin, and stained with hematoxylin and eosin. For immunofluorescence analysis, paraffin sections were incubated with anti-human AIM (AnaSpec, Fremont, CA, USA), anti-human CD14 (Abcam plc, Cambridge, UK), and anti-human CD16 (AbD Serotec, Oxford, UK), followed by imaging under a fluorescence microscope.

### Statistical analysis

Results are expressed as means ± standard deviation (SD). *P* values less than 0.05 were considered statistically significant. Statistical analyses were performed using the Chi-square or Mann-Whitney *U* test, as appropriate. Correlation coefficients were calculated by Spearman’s rank correlation analysis. The discriminatory power for each putative marker was described using the receiver operating characteristics (ROC) area under the curve (AUC) (ROC-AUC). Cut-off values were obtained from ROC-AUC analysis. Statistical analysis was conducted using PASW Statistics 18 (SPSS Inc., Chicago, IL, USA).

## Results

### Serum AIM levels and clinical characteristics in patients with IBD and healthy controls

**Fig. 1:**
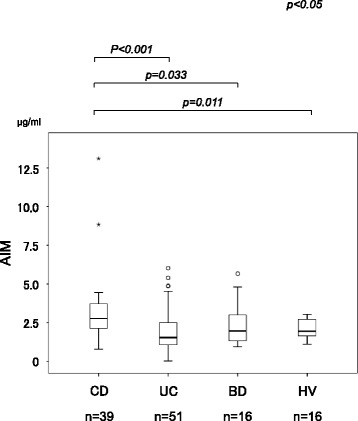
Comparison of serum AIM levels in patients with Crohn’s disease (CD), ulcerative colitis (UC), intestinal Behcet’s disease (BD), and healthy volunteers (HV). Serum AIM levels in the patients with CD were significantly higher than those in the other groups

The clinical characteristics of subjects are summarized in Table 1. The serum AIM concentrations in 39 patients with CD, 51 with UC, 17 with BD, and 16 healthy controls were 3.27 ± 2.14, 1.88 ± 1.43, 2.34 ± 1.37, and 2.13 ± 0.64 μg/ml, respectively (Table 1). Serum AIM levels in patients with CD were significantly higher than those in patients with UC and BD and those in healthy controls (*p* < 0.01 for the other groups); there was no significant difference among the latter 3 groups. Figure 1 shows the serum AIM concentration in each group. There was no significant difference between white blood cell (WBC) counts or levels of AIM, IgM, or CRP among the groups with CD, UC, and BD.

### Association between serum AIM levels and other laboratory data in patients with CD

**Fig. 2:**
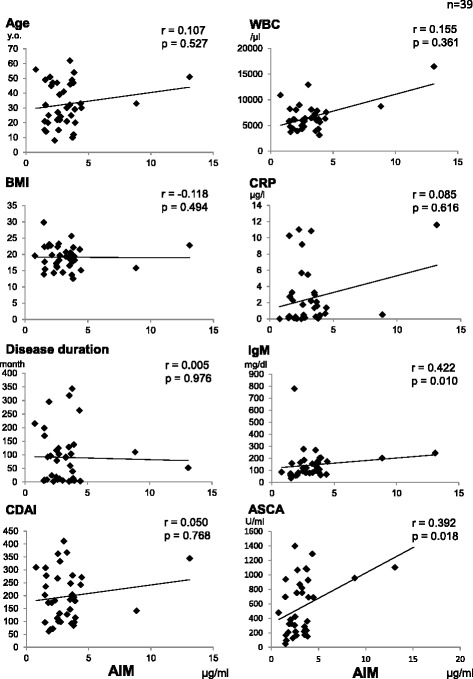
Correlation among serum AIM levels and various *parameters* and *inflammatory markers* in patients with CD. IgM were positively correlated with serum AIM levels. There were no statistically significant differences between *AIM levels* and *other markers*

**Fig. 3:**
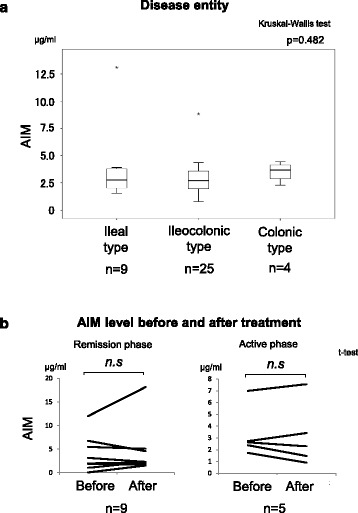
**a**. Comparison of serum AIM levels in patients with CD, UC, or intestinal BD. **b**. Comparison of serum AIM concentrations before and after treatment in patients with CD in active (*n* = 5) and remission (*n* = 9) stages. Induction treatments comprised prednisolone, tacrolimus, 5-ASA, cyclosporine, azathioprine, and/or leukocytapheresis in all 14 patients with CD. Nine patients successfully entered *remission* while five patients remained in the *active* stage after induction treatment. There was no significant difference in AIM levels between the two groups. Each *symbol* indicates one patient

We found that serum AIM levels were positively correlated with IgM in patients with CD. In contrast, no IBD patients showed significant associations between serum AIM levels and inflammatory markers such as WBC counts, CRP levels, age, disease duration, body mass index, or CDAI scores (Fig. 2). In addition, there was no association between AIM levels and disease entities (Fig. 3). We also compared serum AIM levels before and after induction therapy in 14 patients with CD, 9 of whom had successfully entered the remission phase and 5 of whom were in the chronic active phase (Table 2). None of these patients showed an association between serum AIM levels and disease activity (Fig. 3).

**Table 2:** Clinical characteristics of CD patients in remission or active phase

	Remission	Active	*P* value
Number of patients	9	5	
Gender			
Male	3	2	0.969
Female	6	3	
Mean age (±SD), yr	28.8 ± 16.8	24.0 ± 8.7	0.898
BMI (±SD), kg/m^2^	18.6 ± 3.1	20.2 ± 6.3	0.898
CDAI			
≥ 150	5	4	0.658
< 150	4	1	
Disease location			
Ileal type	3	0	0.167
Ileocolonic type	6	4	
Colonic type	0	1	
Induction therapy ^a^			
5-ASA	6	3	
Steroid	1	0	
Azathioprine	2	0	
Infliximab	8	5	
Adalimumab	1	0	
GMA/LCAP	0	0	
AIM (μg/ml)			
Before treatment			
Average (±SD)	3.8 ± 3.7	3.3 ± 2.1	0.797
After treatment			
Average (±SD)	4.4 ± 5.3	3.1 ± 2.7	0.699

^a^some treatments overlapped

### Diagnostic utility of serum AIM and ASCA levels in CD

**Fig. 4:**
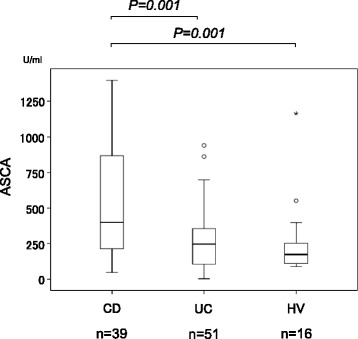
Comparison of serum ASCA levels between patients with Crohn’s disease (CD), ulcerative colitis (UC), and healthy volunteers (HV). Serum AIM levels were significantly *higher* in patients with CD than in the other groups

We next compared serum ASCA-IgG levels to evaluate the utility of AIM for distinguishing between CD and UC. The means ± SD of ASCA were 955.7 ± 382.6, 270.4 ± 205, and 268.5 ± 278.3 U/ml in patients with CD, patients with UC, and healthy controls, respectively. Serum ASCA levels in the CD patients were significantly higher than those in patients with UC and healthy controls (*p* < 0.01 for the other groups; Fig. 4).

**Fig. 5:**
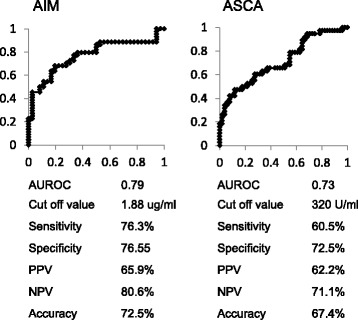
Receiver operating characteristic (ROC) *curves* of apoptosis inhibitor of macrophage (AIM) and anti-Saccharomyces cerevisiae antibodies (ASCA) for the diagnosis of Crohn’s disease (CD). The ROC curves for AIM and ASCA were obtained by *plotting* sensitivity versus 1-specificity. Sensitivity and specificity were each calculated using ELISA. AIM had a sensitivity of 68.2%, a specificity of 80.6%, a *positive* predictive value (PPV) of 65.9%, a *negative* predictive value (NPV) of 80.6%, and an accuracy of 72.5% for distinguishing CD from ulcerative colitis, while the corresponding values for ASCA were 60.5, 72.5, 62.2, 71.1, and 67.4%, respectively

Although, there was association between AIM levels and serum ASCA levels (Fig. 2), the ROC-AUCs of AIM and ASCA were 0.79 and 0.73, respectively. ROC-AUC analysis revealed that a serum AIM level of 1.88 μg/ml was the optimal cut-off value to differentiate between CD and UC, with 76.3% sensitivity and 76.5% specificity, a positive predictive value of 65.9%, a negative predictive value of 80.6, and 72.5% accuracy. With a cut-off value of 320 U/ml, ASCA had a sensitivity of 60.5%, a specificity of 72.5%, a positive predictive value of 62.2%, a negative predictive value of 71.1, and 67.4% accuracy (Fig. 5). Thus, the diagnostic utility of AIM is non-inferior to ASCA for the differential diagnosis of UC and CD.

### AIM expression in intestinal macrophages

**Fig. 6:**
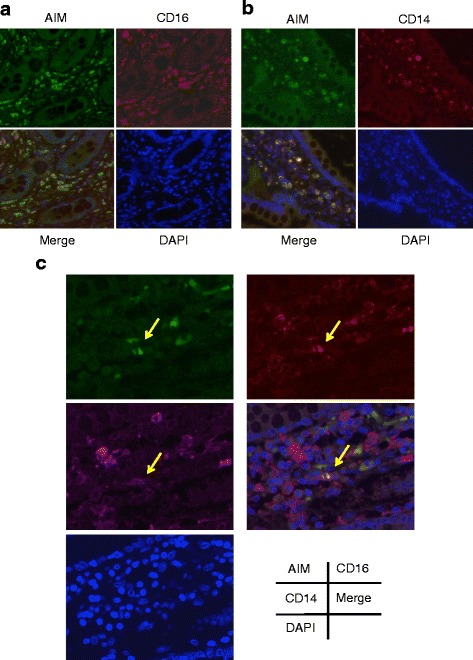
Immunohistochemical staining of small intestine samples from patients with *active* CD. AIM expression is noted in submucosal mononuclear cells *positive* for CD14 (**a**) or CD16 (**b**). Some mononuclear cells were *positive* for CD14, CD16, and AIM antibodies (**c**; *arrow*). Microscopic magnification is × 400

Histological evaluation was performed to localize AIM in tissue sections from patients with CD who had undergone intestinal resection due to severe intestinal inflammation. The small intestinal tissue of patients with CD showed invasion of inflammatory cells into the submucosa (hematoxylin and eosin staining). Immunostaining showed that AIM was strongly expressed in intestinal mononuclear but not epithelial cells in patients with CD (Fig. 6). Double immunostaining, performed to identify cells expressing AIM, showed that a number of AIM-positive mononuclear cells were stained by antibodies to CD14, CD16, or both, and AIM was dominantly expressed in CD16-positive cells. These results demonstrate that AIM is expressed in active macrophages located in the intestinal mucosa (Fig. 6).

## Discussion

This study is the first to measure serum AIM concentrations in patients with IBD. We showed that serum AIM levels were higher in CD patients than in those with UC or intestinal BD or in healthy controls. In addition, we confirmed that AIM was expressed in CD14- and CD16-positive macrophages in intestinal tissue. AIM, a member of the scavenger receptor cysteine-rich superfamily, is initially identified as an apoptosis inhibitor that supports the survival of tissue macrophages against different types of pro-apoptotic stimuli [28, 29]. We speculate that AIM, produced by resident macrophages in intestinal tissue, contributes to intestinal inflammation, and active macrophage-derived AIM in the intestines results in elevated serum AIM levels.

Intestinal macrophages play important roles in local homeostasis and mucosal inflammation. Unique macrophages in normal colonic mucosa generally attenuate immune functions and induce protective immunity. As a result, intestinal macrophages contribute significantly to the pathogenesis of CD [5, 6]. Colonic macrophages show higher expression of CD14 and CD16 in patients with IBD than in healthy controls, indicating additional macrophage populations in the inflamed mucosa [5]. Infiltrating macrophages contribute to the initiation and sustention of the mucosal inflammation that is characteristic of human IBD. In addition, peripheral blood monocytes expressing CD16 were previously identified as a major proinflammatory cell population, producing cytokines such as tumor necrosis factor-α (TNF-α), interleukin (IL)-1, and IL-12 [30, 31]. Koch et al. reported that CD16-positive peripheral blood monocytes were significantly increased in active CD, particularly in patients with colonic involvement. Thus, CD14- and CD16-positive monocytes and macrophages, which are major contributors to the inflammatory infiltrate in the lamina propria, constitute a pivotal proinflammatory cell population in CD due to their production of large amounts of inflammatory cytokines such as IL-12, IL-23, and TNF-α [32–34].

Given the above, a significant reduction in peripheral CD14- and CD16-positive monocytes by leukocyte apheresis/adsorption and corticosteroids should improve intestinal inflammation [34, 35]. Azathioprine was also shown to inhibit the proliferation of CD16-positive cells in blood and lamina, resulting in the improvement of enterocolitis. In addition, infliximab has a reduced effect if there are no CD14-positive cells in the lamina propria. These result show that AIM derived from CD14- or CD16-positive cells inhibits the apoptosis of intestinal macrophages by autocrine or paracrine mechanisms, and may thereby result in the sustentation of intestinal inflammation.

Recent clinical studies have reported high serum AIM levels in patients with liver fibrosis and chronic hepatitis C, [36] liver damage in patients with hepatocellular carcinoma, [37] and obesity-associated autoimmune disease in humans [38]. We confirmed that serum levels of AIM, which is actively secreted by active macrophage in the intestine, were found at higher levels in patients with CD than in patients with UC or intestinal BD or in healthy controls. Since AIM levels were high only in the patients with CD and not in those with UC or intestinal BD, the evaluation of AIM levels may be helpful as an auxiliary method of discriminating between CD and other chronic intestinal diseases. Although serum AIM levels were not associated with disease activity or clinical characteristics in CD, and there were no changes in AIM levels before and after treatment, increases of AIM are likely to be related to the pathogenesis of CD, namely in the sustentation of inflammation due to the inhibition of macrophage apoptosis.

ASCA, a major serum marker, has been reported as a screening tool for CD. In this study, ROC analysis showed that AIM had better sensitivity and specificity than ASCA for the screening of CD and intestinal inflammation disease. It is a well-known fact that ASCA is a less useful test in Asian than Caucasian populations [39]. It is possible that AIM can be used to screen for CD in patients from the Asian general population who have undiagnosed gastrointestinal symptoms.

The sequential assessments of AIM levels in the same CD patients before and after treatment showed no changes in this study. While this analysis comprised only a small number of patients, serum AIM levels do not appear to be associated with disease activity in CD. However it is suggested that the upregulation of AIM may reflect the sustention of chronic inflammation in CD caused by the inhibition of apoptosis of active intestinal macrophages, and correlate with disease susceptibility in CD.

It has long been recognized that hypertrophied mesenteric adipose tissues surround the intestinal mesentery in CD [40]. Although TNF-α derived from these mesenteric adipose tissues induces intestinal inflammation, the cause and pathology of the CD-specific mesenteric adipose tissue accession have not been clearly elucidated. AIM is incorporated into adipocytes via CD36-mediated endocytosis, and subsequently induces lipolysis. Therefore it may play a role in inflammatory macrophage recruitment and induce TNF-α in mesenteric adipose tissues in CD [26–28, 41, 42]. AIM may influence the pathogenesis of CD not only by inhibiting the apoptosis of active intestinal macrophages, but also by enhancing the expression of TNF-α in these mesenteric adipose tissues.

There are several limitations to this study. First, it used a single-center design and enrolled a small number of patients. Therefore we should examine a larger sample in the near future. Second, AIM was expressed by active macrophages, although there was no association between serum AIM levels and CD activity. The inductive activity of AIM expression in macrophages is still unclear in the inflamed intestines of patients with CD. We plan to investigate this issue by analyzing the function of the AIM gene using gene-modified mice, as there have been no reports of colitis mouse models in AIM knockout mice.

## Conclusions

AIM is a novel biomarker of CD that can distinguish CD from UC or BD. It is suggested that AIM may contribute to intestinal inflammation by inhibiting the apoptosis of macrophages.

## Abbreviations

AIM: Apoptosis inhibitor of macrophages
ASCA: Anti-*Saccharomyces cerevisiae* antibodies
AUC: Area under the curve
BD: Behcet’s disease
CAI: Clinical Activity Index
CD: Crohn’s disease
CDAI: Crohn’s Disease Activity Index
CRP: C-reactive protein
ELISA: Enzyme-linked immunosorbent assay
ESR: Erythrocyte sedimentation rate
IBD: Inflammatory bowel disease
IgG: Immunoglobulin G
IgM: Immunoglobulin M
IL: Interleukin
ROC: Receiver operating characteristics
ROC-AUC: Receiver operating characteristics area under the curve
SD: Standard deviation
TNF-α: Tumor necrosis factor-α
UC: Ulcerative colitis
WBC: White blood cell
